# Evaluation and Optimization of Downstream Process Parameters for Extraction of Betulinic Acid from the Bark of *Ziziphus jujubae* L.

**DOI:** 10.1155/2013/469674

**Published:** 2013-11-13

**Authors:** Kashyap Kumar Dubey, Nitika Goel

**Affiliations:** Microbial Biotechnology Laboratory, University Institute of Engineering and Technology, MD University, Rohtak, Haryana 124001, India

## Abstract

Present work investigated an apposite and efficient method for extraction of betulinic acid (BA) from the bark of *Ziziphus jujubae*. Various extraction methods like stirring extraction, soxhlet extraction, ultrasonic extraction, and microwave assisted extraction (MAE) were evaluated for increasing recovery percentage of BA. From the raffinate so obtained, BA was isolated. Thin layer chromatography (TLC) was used to analyze the extract and high performance liquid chromatography (HPLC) for quantification. The results revealed that the percentage extraction of BA from *Z. jujubae* by MAE was more proficient. As recovery percentage of BA by MAE technique turned out to be maximum, by using response surface methodology (RSM), three process parameters (pH, temperature, and time) were optimized by MAE and it was observed that the optimum parameters (pH 6.5, temp. 70.23°C, and time 3.5 min) gave the maximum recovery of BA (0.44% w/w). To validate the RSM model, experiments were performed and the highest recovery of BA was found to be 0.4% w/w which is ±0.04% to the predicted value. Henceforth the extraction efficiency and the substantial saving of time by MAE was more capable than the other extraction techniques.

## 1. Introduction

**Figure 1:**
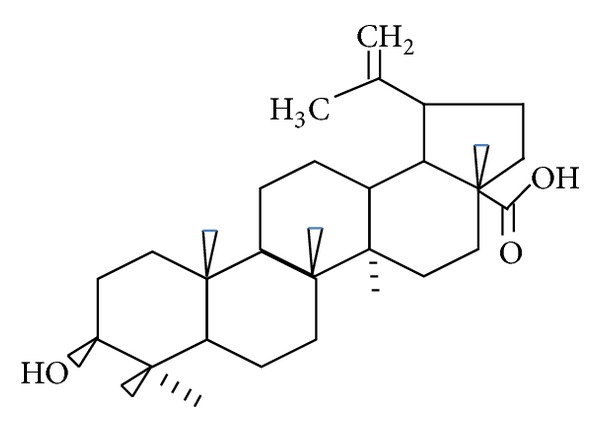
Chemical structure of betulinic acid.

Betulinic acid (3**β**-hydroxy-lup-20-en-28-oic acid) is a naturally occurring pentacyclic lupane-type triterpenoid unveiling a variety of biological and medicinal properties such as inhibition of human immunodeficiency virus (HIV), antibacterial, antimalarial, anti-inflammatory [1], antihelmintic, antinociceptive, anti-HSV-1, and anticancer activities. Betulinic acid acts through inhibition of the enzymes in the arachidonic [2] acid pathway turning out to be a powerful antiphlogistic [3] agent. BA, as shown in Figure 1, ** **induces apoptosis, that is, the controlled death of a cancer cell, butdoes not affect normal cells [4].

Distribution of BA is very wide in the plant kingdom with birch tree (*Betula *spp., Betulaceae) as one of the most extensively stated sources. Also from the various other sources including *Ziziphus *spp. (Rhamnaceae) [5], *Syzygium *spp. (Myrtaceae), *Diospyros *spp. (Ebenaceae), and *Paeonia *spp. (Paeoniaceae), BA can be isolated.

The jujube fruit originated in China and it is known to stimulate the body, increase metabolism, give strength to the heart, and slow down the aging process. It is rich in calcium, protein, and Vitamins C, B1, B2, and A. Jujube encourages cell turnover, improves elasticity and firmness of skin, and reduces the appearance of scars and stretch marks. There are very few plants or trees which are admired by the Chinese as the jujube tree due to its medications [6] resulting from treatment with extracts from the Chinese jujube. Experiments show that *Ziziphus jujubae* possesses immense pharmacological properties like antiaging effects, anti-inflammatory effects, anticancer and antiviral effects, and antiparasitic effects.

Previous reports have shown that the extraction and product recovery are the most imperious steps in the evaluation of the target molecules from various plant parts. Generally the extraction processes consume a lot of time and bulk amount of solvents. Stirring extraction methods [7] are used for the extraction of biological active compounds [8]. Soxhlet extraction technique [9] is applied for various compounds taking more than 24 h for extraction leading to wastage of large amount of solvents. In ultrasonic extraction (USO), high frequency sound [10] is used to interrupt the target compound from the plant materials. USO has been used for the extraction of saponin from ginseng [11] and also other phytochemicals [12] from different parts of plants. Microwave assisted extraction (MAE) system for extraction of biologically active compounds has many advantages over other conventional extraction methods [13] as it requires shorter time, less solvents, higher extraction rate, and better products with lower costs. Plentiful compounds have been extracted using MAE, such as extraction of glycyrrhizic acid from *Glycyrrhiza glabra *root and extraction of artemisinin from *Artemisia annua* [8].

Normally, in the downstream processes, the optimum conditions are determined by keeping one variable parameter and others at constant level, which is a very time consuming process. Box and Wilson [14] devised response surface methodology (RSM), a statistical approach, which turns out to be an effective tool for optimization process to reduce the time involved in performing so many experiments.

The present research aimed to develop a suitable efficient downstream process for the maximum recovery of BA using central composite design (CCD) of RSM to evaluate and understand the process parameters individually as well as their interactions. On the basis of previous reports [7–9, 11], the authors have chosen few extraction procedures, namely, stirring extraction, soxhlet extraction, ultrasonic extraction, and MAE for enhancement in recovery of BA from the bark of *Ziziphus jujubae*.

## 2. Materials and Methods

### 2.1. Plant Material and Reagents

Plant material of *Z. jujubae *was provided by Sai Phytoceuticals, New Delhi, India. The bark was dried at 55°C in air dryer for 45 h. Dried material was pulverized by miller. Ethanol, methanol, hexane, acetone, and ethyl acetate were of analytical grade chemicals (SISCO Research Lab, Mumbai, India). Petroleum ether, dichloroethylene, and acetic acid were used for TLC (Merck, Mumbai, India). Acetonitrile and water were used for HPLC of reagent of HPLC grade (SRL, Mumbai, India).

### 2.2. Extraction Procedures and Related Parameters

Four types of extraction procedures, namely, stirring extraction, ultrasonic extraction, soxhlet extraction, and microwave assisted extraction, were tried. Parameters that were optimized were choice of solvent, material : solvent ratio, pH, process time, and temperature for the maximum recovery of BA from the bark of *Z. jujubae*. The procedures for the optimization of the above described parameters using different extraction techniques are as follows.

**Figure 2:**
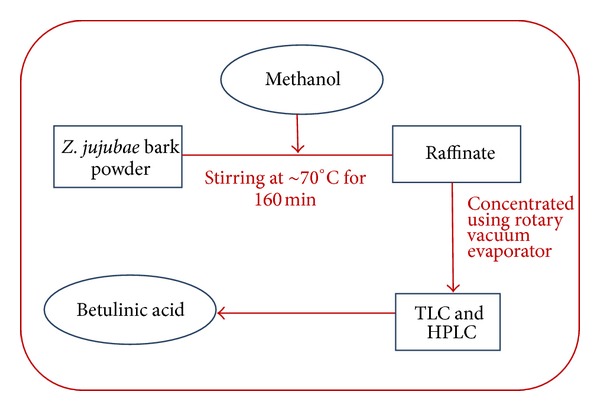
Line diagram for stirring extraction method.

Stirring extraction was carried out on a magnetic stirrer furnished with a hot plate. Ground bark material of *Z. jujubae* was extracted with ethanol, methanol, hexane, acetone, and ethyl acetate in similar concentrations at 70°C for 160 min (Figure 2) successively at a speed of 160 ×g. After extraction, the raffinates were filtered through filter paper and vacuum dried using rotary vacuum evaporator. The filtrates were isolated by TLC and subsequently analyzed by HPLC. The stirring extraction procedure was repeated with methanol at different pH and varying bark material : methanol ratios.

**Figure 3:**
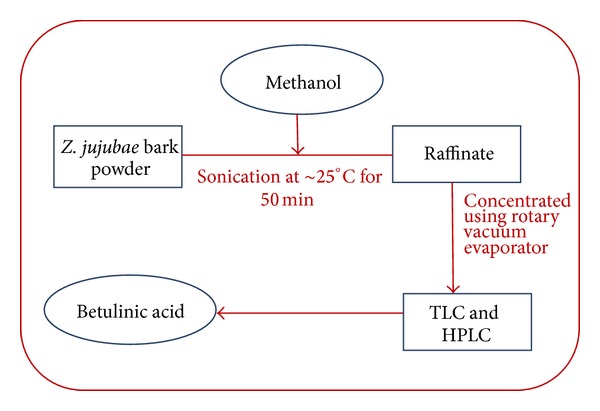
Line diagram for ultrasonic assisted extraction method.

Ultrasound assisted extraction was accomplished in an ultrasonic bath with a working frequency of 35 KHz. Two-gram *Z. jujubae* plant material (Figure 3) was extracted with methanol (100 mL) and kept for sonication for 20, 30, 40, 50, and 60 min at room temperature sequentially.

**Figure 4:**
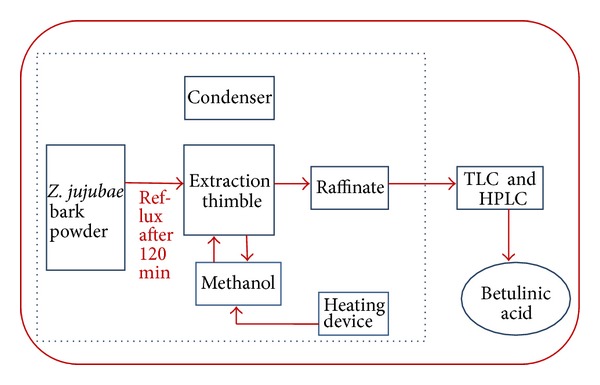
Line diagram for soxhlet extraction method.

During soxhlet extraction (Figure 4), in the 200 mL soxhlet thimble, 2 g of dried bark powder was set and the apparatus was fixed with a round bottom flask holding 100 mL of methanol. The extraction was carried out at 68°C for 40, 60, 80, 100, 120, and 140 min, and after a given time, the solvent was refluxed.

**Figure 5:**
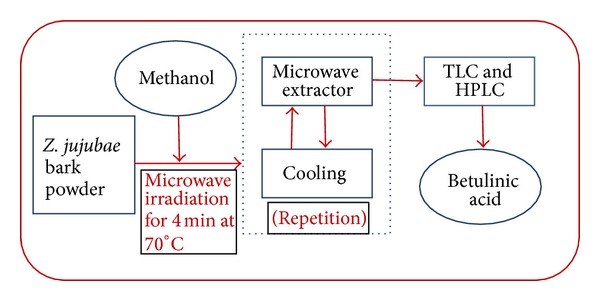
Line diagram for microwave assisted extraction (MAE) method.

For microwave assisted extraction, *Z. jujubae *dried bark powder (2 g) was put into a 100 mL flask to which 100 mL of methanol was added. The flask was then exposed for 4 min in a microwave oven at 100 W. Superboiling was not allowed as the microwave irradiation was stopped for a minute and was cooled underneath running water for 2 min, and then the same flask was exposed to microwave irradiation (Figure 5) after cooling.

### 2.3. Optimization of Process Parameters in MAE Using RSM

The three most significant factors (*A*: pH, *B*: time, and *C*: temperature) in MAE technique, affecting the extraction rate of BA, were optimized using CCD. To produce and examine the experimental design, the statistical software “Design Expert 8.0” was employed. A set of 20 different runs were carried out and the maximum recovery % of BA (w/w) was used as response on accomplishment of the experiments. To obtain an empirical model, linking the response measured with the independent variables, multiple regression of the data was also carried out. Using the “Design Expert” software, 3D graphs were formed to define the optimum levels of the variables for maximum yield of BA.  Table 1 shows the experimental range for each variable using the classical optimization approach where one parameter was changed while others were kept constant.

### 2.4. Chromatography Analysis

The raffinates obtained from different extraction methods were filtered and concentrated by rotary vacuum evaporator and then BA was isolated using TLC. TLC was performed in silica gel as a stationary phase and the solvents used as mobile phase were petroleum : dichloroethylene : acetic acid (50 : 50 : 0.7). The betulinic acid was detected by spraying 20% antimony chloride in chloroform.

The HPLC analyses were carried out on furnished YL instrument. The betulinic acid was determined by using 84% acetonitrile and 16% water as mobile phase. The flow rate was 1 mL/min and the elution was monitored at 210 nm. Validation of quantitative method was done with sample three times.

## 3. Results and Discussion

**Figure 6:**
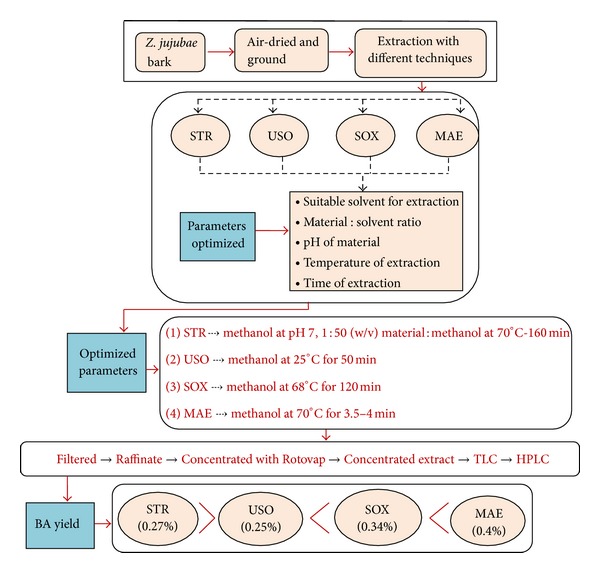
Representation of the complete process for recovery of BA from the bark of *Z. jujubae *(STR: stirring extraction; USO: ultrasonic extraction; SOX: soxhlet extraction; MAE: microwave assisted extraction).

Betulinic acid is a pentacyclic lupane-type triterpenoid which exhibits various biological and medicinal properties such as antibacterial, antimalarial, antiinflammatory, anthelmintic, antinociceptive, anti-HSV-1, and anticancer activities [1, 3]. Previous researchers have been exploiting stirring extraction, ultrasonic extraction, soxhlet extraction, and MAE for extraction of BA. In the present study, the authors have investigated suitable techniques among the above mentioned methods for the recovery of BA from the bark of *Z. jujubae.* Figure 6 depicts the line diagram of downstream process for the recovery of BA from the bark of *Z. jujubae*.

The extraction parameters were optimized and the results are given below.

### 3.1. Selection of an Appropriate Solvent

**Figure 7:**
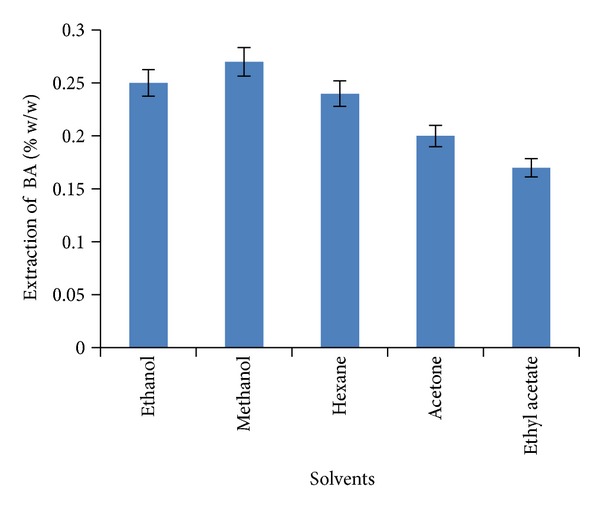
The effect of stirring extraction with various solvents at 70°C for 160 min.

Various organic solvents (methanol, ethanol, acetone, ethyl acetate, and hexane) were used in the extraction of BA from the bark of *Z. jujubae*. The maximum recovery of BA (0.27% w/w) was obtained when methanol was used as organic solvent as compared to the other solvent systems (Figure 7). Previous literature reported that methanol was found to be most reactive with the plant material during extraction of secondary metabolites [15] especially terpenes. Generally, methanol was regarded as a safe solvent system for extraction of medicinally important metabolites. Keeping in view the above facts, methanol was used as a solvent for further extraction procedures.

### 3.2. Optimization of Material : Solvent Ratio

**Figure 8:**
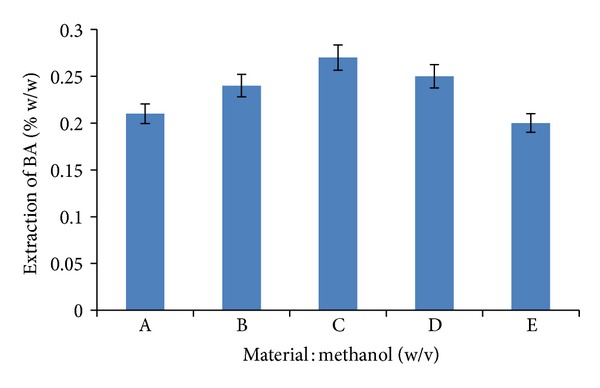
The effect of stirring extraction at varying material : solvent ratios (*A* = 1 : 30, *B* = 1 : 40, *C* = 1 : 50, *D* = 1 : 60, and *E* = 1 : 70).

To minimize the cost of the recovery process several material : solvent ratios were tried. The different material : solvent (methanol) ratios were 1 : 30, 1 : 40, 1 : 50, 1 : 60, and 1 : 70 at lab-scale level.  Figure 8 shows that the 1 : 50 material : methanol ratio gave the maximum recovery (0.27% w/w) of BA. So, further in the study, 1 : 50 ratio was used for the extraction of BA. Hence, to reduce the excess solvent usage, 1 : 50 material : methanol ratio was used in USO, SOX, and MAE techniques.

### 3.3. Optimization of pH of Material (Plant Bark)

**Figure 9:**
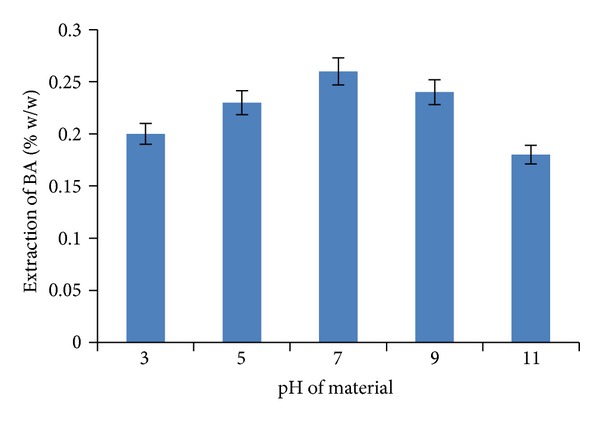
The effect of stirring extraction with methanol at varying pH values.

In order to obtain the maximum recovery of BA from the bark of *Z. jujubae* with methanol as a solvent, extractions were carried out at differing pH (3, 5, 7, 9, and 11) values. Results unveil that the maximum recovery of BA (0.26% w/w) was seen at pH 7. Similar reports have already been done by previous researchers on other plant secondary metabolites [16]. As shown in Figure 9, the extraction yield of BA increased as the pH values increased from 3 to 7 but decreased at pH values higher than 7 because the covalent bonding between plant material and the solvent becomes weak at pH higher than 7. When pH value is increased from 3 to 7, the extraction yield of BA increases due to the fact that stability of the extracted BA is maintained and is the highest at pH 7, and at pH values above 7, the reduction in the stability of the extracted compound, that is, BA, is observed.

### 3.4. Process Time Optimization

**Figure 10:**
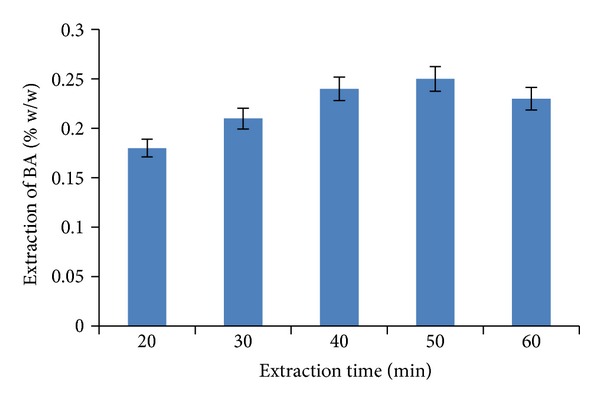
The effect of ultrasonic extraction time on percentage extraction of BA.

Optimization of the process time for the maximum recovery of BA from the plant material depended upon the type of extraction technique used. In stirring extraction technique, the efficiency of methanol used for maximum extraction was at 160 min yielding 0.27% w/w of BA.  Figure 10 shows that, for USO extraction, the maximum recovery of BA (0.25% w/w) was obtained when the bark material was extracted in sonicator for 50 min.

**Figure 11:**
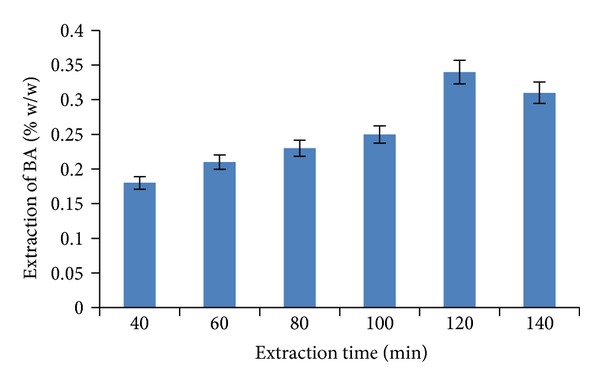
The effect of soxhlet extraction time on percentage extraction of BA.

For SOX extraction, the recovery of BA (Figure 11) was found to be maximum (0.34% w/w) when the material was extracted with methanol for 120 min. As the process time was increased to 140 min, the percentage extraction of BA decreased due to partial breakdown of the material.

To obtain maximum recovery of BA (0.39% w/w) using MAE, the exposure time of flask containing bark powder and methanol for microwave irradiation was found to be 4 min. Additionally, the authors are also concerned about the cost effectiveness of the extraction process so MAE turned out to be the apt extraction technique for the extraction of BA, as it is the quickest requiring the least extraction time. The recovery of BA was significantly high when MAE was used as compared to SOX, STR, and USO extraction techniques.

### 3.5. Optimization of the Process Temperature

**Figure 12:**
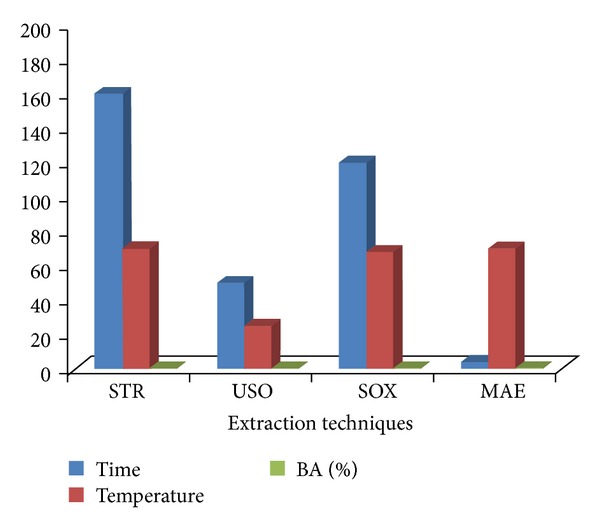
Comparison of percentage extraction of BA from bark of *Z. jujubae *by different extraction techniques.

As the boiling point of methanol is ~65°C, the maximum recovery of BA was obtained within the range of 64–70°C for all types of extraction techniques excluding USO extraction in which maximum recovery was obtained at room temperature.Figure 12 shows that process temperatures at which the percentage extraction of BA was maximum using various extraction techniques were found to be 70°C (0.27% w/w), 25°C (0.25% w/w), 68°C (0.34% w/w), and 70°C (0.39% w/w) for STR, USO, SOX, and MAE techniques, respectively.

### 3.6. Selection of the Most Efficient Extraction Technique

Using the optimized parameters described above, four types of extraction techniques, namely, stirring extraction, ultrasonic extraction, soxhlet extraction, and microwave assisted extraction, were tried to attain the maximum recovery of betulinic acid from the dried and pulverized bark powder of *Z. jujubae*.

In stirring extraction technique, with methanol as a solvent at 70°C for 160 min, 0.27% w/w of BA was obtained. For ultrasonic assisted extraction, the maximum percentage extraction of BA (0.25% w/w) was recovered at 25°C with the process time of 50 min. In order to obtain maximum recovery of BA (0.34% w/w) with soxhlet extraction, the bark material was extracted with methanol at 68°C for 120 min. Figure 12 unveils that, by using MAE, the extraction % of BA was found to be maximum (0.39% w/w) when the material was irradiated for 4 min at 70°C.

Thus microwave assisted extraction turns out to be the most appropriate, the quickest, and the most proficient technique for the maximum recovery of betulinic acid from the bark of *Z. jujubae.*

### 3.7. Optimization of MAE Extraction Using RSM

As explained above (Section 3.6), MAE extraction technique turns out to be the most proficient technique, the three significant factors affecting BA recovery (*A*: pH, *B*: time, and *C*: temperature) were optimized using RSM in MAE extraction method. 

The predicted and observed responses in terms of % recovery of BA (w/w) have been shown in Table 2. The level of BA recovery was given by the second-order regression equation (1), attained after ANOVA in terms of actual factors (Table 3).

BA %yield (w/w):

(1) Y=0.261093+0.006157A+0.30188B +0.104609C−0.0075AB −0.0075AC−0.0125BC −0.05518A2−0.03043B2 +0.004926C2±0.047,

where *Y* is the response symbolizing the yield % of BA (w/w) and *A*, *B*, and *C* are coded values of pH, process time, and temperature.

The coefficient of regression (*R* ^2^) was found to be 0.9059 for BA recovery. This specifies confidence of >95% in the results signifying precise dependence between experimental and predicted values. The experimental value of *R* ^2^ > 0.9059 indicates rightness of the model. To define the experimental factors producing signals that are large in assessment to the noise, statistical analysis is needed. The appropriate precision measures signal-to-noise ratio and a value >4 is desirable, so the adequate precision value of 12.993 for BA recovery specified the usage of model for directing the design space. Analysis of variance for the betulinic acid extracted from *Z. jujubae *roots obtained from this design was given in Table 3.

**Figure 13:**
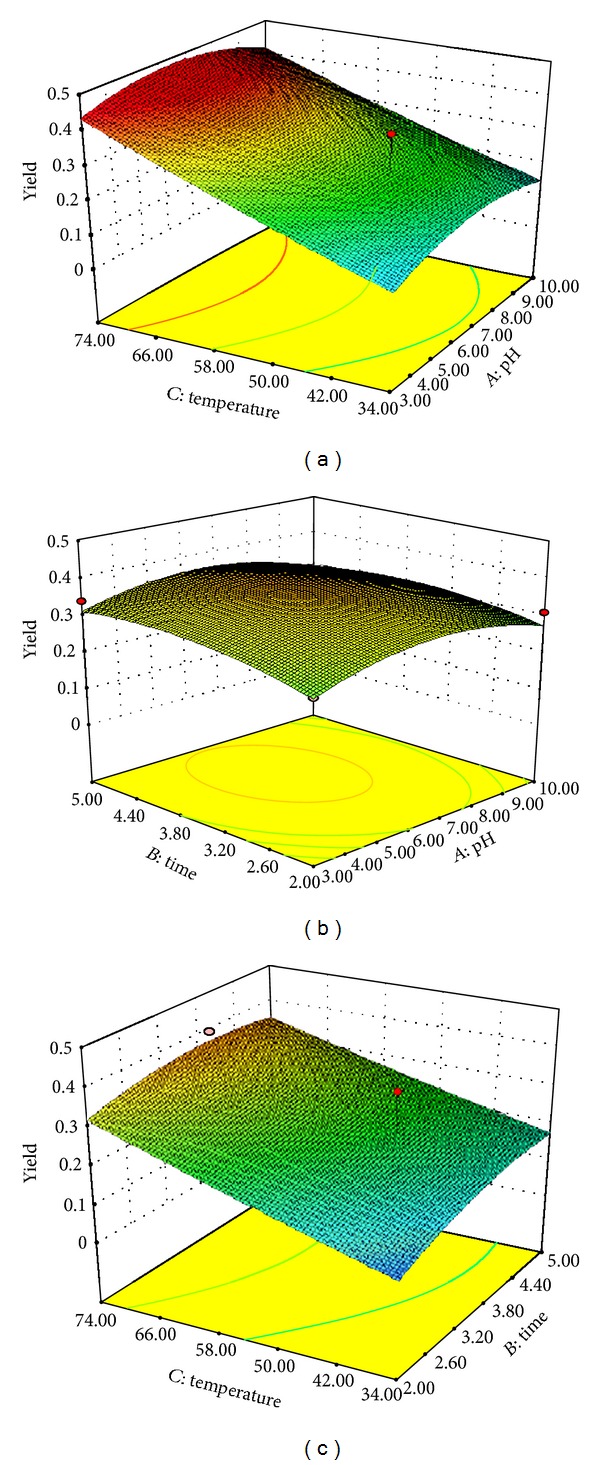
(a) Effect of pH and temperature on the yield of BA in the extraction process: 3D plot shows that % BA yield (w/w) increases with increase in pH and extraction temperature. The third factor (process time) is fixed at 3.5 min. (b) Effect of process time and pH on the BA yield in the extraction process: 3D plot showing increase in % BA yield (w/w) with increase in pH and process time. The third factor (temperature) was fixed at 70.23°C. (c) Effect of process time and temperature on the BA yield in the extraction process: 3D plot showing increase in % BA yield (w/w) with increase in process time and extraction temperature. The third factor (pH) is fixed at 6.5.

The model *F*-value and *P* value of 10.7 and 0.0005 for BA recovery unveiled that the model is significant (Table 3). Values of “Prob. > *F*” less than 0.0500 show model terms which are significant. The “lack of fit *F*-value” of 0.62 implied the lack of fit is not significant relative to the pure error. 3D graphs define the relations of the variables and also the optimum level of each variable for maximum BA recovery (Figures 13(a), 13(b), and 13(c)). The model prediction for maximum BA recovery (0.44% w/w) was at temperature 70.23°C, pH 6.5, and process time of 3.5 min.

### 3.8. Evaluation of the Model

At the optimum levels of pH: 6.5, time: 3.5 min, and temp.: 70.23°C, as predicted by RSM, the experimental response for BA recovery was 0.4% (w/w) quantified by HPLC which is ±0.04% to the predicted response of 0.44% (w/w). Hence the statistical model was successfully evaluated. It was observed that process time and temperature conditions for extraction processes play a major role in maximum recovery of BA from *Ziziphus jujubae* which is related to the extraction of triterpenoid, oleanolic acid, from *Lantana camara* [17]. Recovery of BA increased when pH was increased from 3 to 6 and was maximum at pH 6.5 but further increase in pH decreased the BA yield due to the fact that the covalent bonding between plant material and the solvent becomes weak at pH higher than 7. Similarly with increase in process time and temperature conditions, the % BA yield (w/w) also increased as shown in Figure 13. Therefore it was seen that the highest recovery of BA (0.4% w/w) from *Ziziphus jujubae *was obtained using MAE technique at pH 6.5, process time of 3.5 min, and at 70.23°C temperature.

## 4. Conclusion

By assessing various extraction methods for extraction of betulinic acid, MAE was the most resourceful. For product recovery with solvents of ethanol, methanol, hexane, acetone, and ethyl acetate, methanol obtained maximum recovery percentage of BA (0.39% w/w), using MAE, at pH 7 when 1 : 50 bark powder : methanol ratio was used. The extraction temperatures and the time required for the highest extraction percentage of BA for different techniques, namely, stirring extraction, ultrasonic extraction, soxhlet extraction, and MAE, were found to be 70°C, 160 min; 25°C, 50 min; 68°C, 120 min; and 70°C, 4 min, respectively, yielding 0.27%, 0.25%, 0.34%, and 0.39% w/w of BA correspondingly. When RSM was employed in MAE, it predicted the maximum recovery of BA (0.44% w/w) which is ±0.04% to the experimental BA yield (0.4% w/w) at the optimum parameters of pH: 6.5; process time: 3.5 min; and temperature: 70.23°C. Therefore, MAE was found to be the most efficient extraction technique for rapid and maximum percentage extraction of betulinic acid from the dried bark material of *Ziziphus jujubae*.

## Figures and Tables

**Table 1:** Ranges of various independent process variables (temperature, pH, and process time) used in RSM.

Factor	Name	Range studied	
		Low level	High level
*A*	pH	3.00	10.00
*B*	Time	2.00	5.00
*C*	Temp.	30.00	60.00

**Table 2:** Design matrix for the optimization of extraction parameters for maximum recovery of betulinic acid as response.

Run	Type	Factors			BA yield (% w/w)	
		pH	Time (min)	Temp. (°C)	Observed	Predicted
1	Center	6.50	3.50	45.00	0.21	0.24
2	Center	6.50	3.50	45.00	0.21	0.24
7	Center	6.50	3.50	45.00	0.21	0.24
8	Center	6.50	3.50	45.00	0.21	0.24
16	Center	6.50	3.50	45.00	0.35	0.37
19	Center	6.50	3.50	45.00	0.21	0.24
3	Axial	6.50	3.50	70.23	0.4	0.44
6	Axial	0.61	3.50	45.00	0.06	0.07
15	Axial	12.39	3.50	45.00	0.1	0.12
17	Axial	6.50	3.50	19.77	0.08	0.09
18	Axial	6.50	6.02	45.00	0.21	0.24
20	Axial	6.50	0.98	45.00	0.07	0.09
4	Factorial	10.00	5.00	60.00	0.25	0.26
5	Factorial	3.00	5.00	30.00	0.06	0.09
9	Factorial	10.00	5.00	30.00	0.12	0.14
10	Factorial	3.00	2.00	60.00	0.24	0.26
11	Factorial	10.00	2.00	60.00	0.29	0.31
12	Factorial	3.00	5.00	60.00	0.33	0.34
13	Factorial	3.00	2.00	30.00	0.04	0.06
14	Factorial	10.00	2.00	30.00	0.05	0.04

**Table 3:** ANOVA for response surface quadratic model to verify whether developed model is significant or nonsignificant.

Source	Sum of squares	Degree of freedom	Mean square	*F*-value	*P* value Prob. > *F*	
Model	0.21985	9	0.024	10.6998	0.0005	Significant
*A*-pH	0.00052	1	0.0005	0.22679	0.6441	
*B*-time	0.01245	1	0.012	5.45133	0.0417	
*C*-temp.	0.14945	1	0.149	65.4605	<0.0001	
*AB*	0.00045	1	0.0004	0.19711	0.6665	
*AC*	0.0045	1	0.0004	0.19711	0.6665	
*BC*	0.00125	1	0.0012	0.54752	0.4763	
*A* ^2^	0.04388	1	0.044	19.2191	0.0014	
*B* ^2^	0.01334	1	0.013	5.84505	0.0362	
*C* ^2^	0.00035	1	0.0003	0.15316	0.7037	
Residual	0.02283	10	0.0022			
Lack of fit	0.00875	5	0.0017	0.62107	0.6930	Nonsignificant
